# Supplementary material for: Epidemiological characteristics of hand, foot and mouth disease in Pingdu, Shandong, China from 2013 to 2023 and DLNM analysis of its variation with temperature
Source: PLoS One. 2025 Oct 24;20(10):e0333124. doi: 10.1371/journal.pone.0333124 (PMC12551877; doi:10.1371/journal.pone.0333124)
Supplement: Attachment — Submitted filename: response.docx [file pone.0333124.s005.docx]

Dear reviewers，

The main corrections in the paper and the responds to the reviewer’s comments are as flowing:

Journal Requirements:

1. Please ensure that your manuscript meets PLOS ONE's style requirements, including those for file naming.

Author response: We have carefully reviewed and revised our manuscript to comply with PLOS ONE's style requirements, including file naming conventions as outlined in the provided templates.

2. Clarification on the data availability statement and sharing plan.

Author response: Data Availability Statement: All data generated or analyzed during this study are included in this published article. The dataset (the daily number of HFMD cases from 2013 to 2023) used and/or analyzed during the current study were obtained from Pingdu Center for Disease Control and Prevention, Qingdao, China. We have no right to make this dataset public. If you need the dataset, you can contact with the organization (Tel: +86-532-88328906; Address: NO.222 Changzhou Road, Pingdu, Qingdao, People’s republic of China, 266700) (L301-307)

3. Referencing Figures 3, 4, 5, and 6 in the text.

Author response: We have ensured that Figures 3, 4, 5, and 6 are appropriately referenced in the text to facilitate linking for readers upon acceptance.

4. Review and evaluate specific previously published works suggested by reviewers.

Author response: None of reviewers suggested any publication to cite.

5. Review the reference list for completeness and correctness.
Author response: We have thoroughly reviewed our reference list to ensure it is complete and correct, without retracted papers.

Response to reviewers

#1 reviewer

1. The article is not conclusive despite having interesting and conclusive information in the discussion section.

Author response: We appreciate your feedback regarding the conclusiveness of the article. We have revised the discussion section to enhance clarity and emphasize the key findings more distinctly to provide a stronger conclusion. (L265-268)

2. Statistical analysis is included in the article.

Author response: Thank you for confirming that the statistical analysis is adequately included in the manuscript.

3. Allowed data included, and the author mentioned the limitation.

Author response: We acknowledge your comment. Since the daily incidence data involves sensitive information, it cannot be made public. All other data are available for public access.

4. The article is presentable.
Author response: We appreciate your positive feedback on the presentation of the article.

#2 reviewer

1. The draft needs minor writing improvement.

Author response: We have carefully reviewed the manuscript for language clarity and made necessary revisions to improve the overall writing quality.

2. Data shown in the table can be plotted in histograms.

Author response: Thank you very much for your constructive comments. However, if the data in Table 1 were changed to a histogram, a great deal of information would be lost. Considering the overall structure of the article, we have not modified Table 1.

3. Please include vaccines currently in use against the disease (include trade name, vendor, type of vaccine).

Author response: We have included a section in the manuscript detailing the vaccines currently in use against hand, foot, and mouth disease, along with their vendors, and types. (L222-224)

4. If possible, include loopholes in present disease control measures.

Author response: We have added a discussion on the existing loopholes in disease control measures to provide a more comprehensive view of the current situation. (L258-259)

5. Mention whether the observed correlation of HFMD with temperature is also seen with other viral infections in the region.

Author response: We have included a comparative analysis in the revised manuscript discussing the correlation of HFMD with temperature in relation to other viral infections in the region. (L250-252)

6. It would be nice to explain how a rise in temperature leads to a rise in infection if possible.

Author response: We have expanded the discussion to include potential mechanisms by which rising temperatures may contribute to increased HFMD infections, thereby providing a clearer understanding of this relationship. (L237-239)

We would like to express our great appreciation to you and reviewers for comments on our paper. Looking forward to hearing from you.

Thank you and best regards.

Yours sincerely,

Hua Zhang

Corresponding author: Xiaolin Liu

E-mail: sjobshuier@163.com
